# Nutritional upgrading for omnivorous carpenter ants by the endosymbiont *Blochmannia*

**DOI:** 10.1186/1741-7007-5-48

**Published:** 2007-10-30

**Authors:** Heike Feldhaar, Josef Straka, Markus Krischke, Kristina Berthold, Sascha Stoll, Martin J Mueller, Roy Gross

**Affiliations:** 1 Department of Behavioural Physiology and Sociobiology (Zoology II), Biocenter, University of Wuerzburg, Am Hubland, 97074 Wuerzburg, Germany; 2 Department of Pharmaceutical Biology, Julius-von-Sachs-Institute for Biosciences, Biocenter, University of Wuerzburg, Julius-von-Sachs-Platz 2, 97082 Wuerzburg, Germany; 3 Department of Microbiology, Biocenter, University of Wuerzburg, Am Hubland, 97074 Würzburg, Germany

## Abstract

**Background:**

Carpenter ants (genus *Camponotus*) are considered to be omnivores. Nonetheless, the genome sequence of *Blochmannia floridanus*, the obligate intracellular endosymbiont of *Camponotus floridanus*, suggests a function in nutritional upgrading of host resources by the bacterium. Thus, the strongly reduced genome of the endosymbiont retains genes for all subunits of a functional urease, as well as those for biosynthetic pathways for all but one (arginine) of the amino acids essential to the host.

**Results:**

Nutritional upgrading by *Blochmannia *was tested in 90-day feeding experiments with brood-raising in worker-groups on chemically defined diets with and without essential amino acids and treated or not with antibiotics. Control groups were fed with cockroaches, honey water and Bhatkar agar. Worker-groups were provided with brood collected from the queenright mother-colonies (45 eggs and 45 first instar larvae each). Brood production did not differ significantly between groups of symbiotic workers on diets with and without essential amino acids. However, aposymbiotic worker groups raised significantly less brood on a diet lacking essential amino acids. Reduced brood production by aposymbiotic workers was compensated when those groups were provided with essential amino acids in their diet. Decrease of endosymbionts due to treatment with antibiotic was monitored by qRT-PCR and FISH after the 90-day experimental period. Urease function was confirmed by feeding experiments using ^15^N-labelled urea. GC-MS analysis of ^15^N-enrichment of free amino acids in workers revealed significant labelling of the non-essential amino acids alanine, glycine, aspartic acid, and glutamic acid, as well as of the essential amino acids methionine and phenylalanine.

**Conclusion:**

Our results show that endosymbiotic *Blochmannia *nutritionally upgrade the diet of *C. floridanus *hosts to provide essential amino acids, and that it may also play a role in nitrogen recycling via its functional urease. *Blochmannia *may confer a significant fitness advantage via nutritional upgrading by enhancing competitive ability of *Camponotus *with other ant species lacking such an endosymbiont. Domestication of the endosymbiont may have facilitated the evolutionary success of the genus *Camponotus*.

## Background

Insects are among the most successful animal taxa in respect to species richness as well as abundance. Their evolutionary success is in part facilitated by obligate intracellular bacterial endosymbionts that enable some insect groups to live on nutritionally deficient diets and thus in ecological niches that may otherwise be unavailable. Buchner [1] estimated that approximately 20% of all insects harbour intracellular endosymbiotic bacteria. Such insect hosts are usually food specialists [2] with the bacteria supplying essential nutrients that are deficient in the host's diet. For example, aphids that feed exclusively on phloem sap are provided with essential amino acids by *Buchnera *[3], and *Wigglesworthia *supplies blood-feeding tsetse flies with certain vitamins that are deficient in the diet [4,5]. In these ways, endosymbionts upgrade host nutrition by utilizing constituents of the host's food to synthesize compounds that are of higher nutritional value to the host. Usually, either non-essential food constituents are transformed into compounds essential to the host, or compounds that cannot or are poorly metabolized by the host itself are utilized by the bacteria. Although less well documented, endosymbionts may also facilitate life in nutrient-limited niches for insects that are more generalist feeders. In this case endosymbionts may be key to opening new ecological niches for their hosts by contributing nutrients that are not lacking entirely but rather limiting for their hosts.

Carpenter ants (genus *Camponotus*) harbour the obligate intracellular endosymbiont *Blochmannia *in bacteriocytes, intercalated between midgut cells, as well as in ovaries of females [6,7]. With more than 1000 species, *Camponotus *is among the most species-rich and successful genera of ants [8], and is represented in most terrestrial habitats, including among dominant ants of tropical rain forest canopies [9,10]. Suggesting functional importance for the genus as a whole, *Blochmannia *has been detected in all *Camponotus *species investigated to date (> 30 species), including taxa from various different parts of the genus [7,8,11,12]. In addition to *Camponotus*, the bacteria were recently also identified in the related genera *Polyrhachis*, and *Echinopla*, all belonging to the subfamily Formincinae, tribe Camponotini [11,13] and Feldhaar, unpublished results, for ~16 species of *Polyrhachis*). The appearance of *Blochmannia *in closely related genera within the ant subfamily Formicinae suggests an age of the endosymbiosis of approx. 30 to 40 MYA (million years ago) [13,14]. If individuals of every species of these genera contain *Blochmannia*, then approximately 15% of all described ant species today would harbour this obligate endosymbiont [8].

The metabolic capacities of *Blochmannia floridanus *and *Blochmannia pennsylvanicus*, the two endosymbiont species sequenced so far, show remarkable similarity to that of *Buchnera*, the aphid endosymbiont, in their genetic make-up. The genome of *Blochmannia *is strongly reduced in size in comparison to those of its free-living ancestors [15,16] and strongly adenine-thymine-biased. Despite such strong reduction in genome size, biosynthetic pathways for essential amino acids (except arginine) are retained, while those for several non-essential amino acids have been lost [15-17]. *Blochmannia *can apparently synthesize tyrosine, a non-essential amino acid that, together with the essential phenylalanine, is important for tanning and sclerotization of insect cuticle [18]. Accordingly, upregulation of tyrosine biosynthesis genes of the endosymbiont was detected during the pupal stage of hosts and *Blochmannia *may thus supplement the host with tyrosine [19]. *Blochmannia *may also play a role in sulfate reduction to sulfide, a form of sulfur that can be incorporated into biomolecules [17]. Insects themselves are generally unable to reduce oxidized sulfur compounds [20]. Additionally, the ant-endosymbiont contains a complete urease gene cluster. This enzyme hydrolyzes urea to produce CO_2 _and ammonia, and ammonia can then be recycled into the host's amino acid metabolism by the activity of glutamine synthetase, also encoded by *Blochmannia *[17]. In some pathogenic microorganisms, ureases have been identified as important virulence factors [21,22] whereas the urease may be beneficial for the host in this symbiotic association. These metabolic capabilities strongly suggest a role in nutritional upgrading by *Blochmannia *for the ant host.

The aim of this study is to gain insight into the mechanisms of the endosymbiont-host interaction by testing *in vivo *functions predicted from *Blochmannia*'s genome. Genome sequence alone is not an infallible predictor of the endosymbiont's role in symbioses; for example, the same strain of the obligate intracellular bacterium *Wolbachia *confers different fitness benefits depending on its host's genetic background [23]. In addition, for endosymbionts to be involved in interactions with the host, the respective genes must be functional. Endosymbiont genes not under stabilizing selection should deteriorate, and even those needed by insect hosts may be lost, possibly leading to a loss in the symbiotic function of the endosymbiont [24]. This process starts with gene inactivation producing a pseudogene, and only then are major parts of the gene lost from the genome, a process that may take over several million years [25,26]. It is therefore not clear whether an endosymbiont of an omnivore such as *Blochmannia *is still functional as a mutualist or rather a relic of a former mutualistic interaction.

The general relevance of *Blochmannia *for the ant host is apparent from fostering experiments showing that colonies of *C. floridanus *suffer serious fitness losses when workers are cured of infection with *Blochmannia*. When treated with antibiotics, worker groups exhibit significantly reduced success in brood rearing when compared to untreated control groups [19], and larval development may even be arrested entirely [27].

To specifically test whether *Blochmannia *affects host metabolism, we developed a chemically defined diet that allows for omission of specific nutrients [28]. We then tested the hypothesis that *Blochmannia *provides the host with essential amino acids. In a second set of experiments, we traced heavy nitrogen from ^15^N-labelled urea to test whether the urease of the endosymbiont plays a role in nitrogen recycling for the host.

## Results

### Feeding experiments with chemically defined diets

Six colony fragments from each of eight colonies were fed different diets over a 12-week period and monitored for numbers of pupae raised from a total of 45 eggs and 45 first instar larvae supplied to each group.

**Figure 1:**
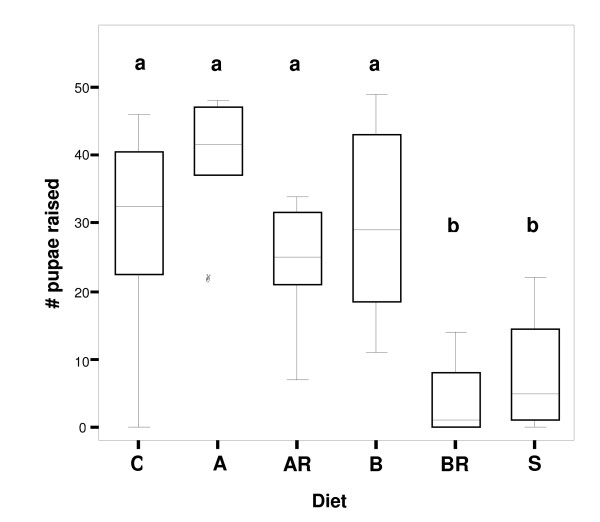
**Number of pupae raised by worker groups fed different diets over a 12-week period**. The maximal possible number of pupae is 90. Differences across diets were significant (ANOVA: p < 0.001, F_5,42 _= 13.338; see Results). Groups differing significantly from each other in Tukey HSD posthoc test are marked with different letters above box-plots. Medians are given in box-plots, and an outlier is marked with a circle. Diets: C = control (Bhatkar agar and honey water), A = artificial diet with essential amino acids, AR = artificial diet plus antibiotic Rifampicin, B = artificial diet without essential amino acids, BR = artificial diet without essential amino acids but with antibiotic Rifampicin, S = sucrose solution only (20% w/v).

Production of pupae was pooled for each set of colony fragments fed the same diet (n = 8), because colony identity did not significantly affect the number of pupae raised (ANOVA: p = 0.199, F_7,40 _= 1.488). Numbers of pupae raised per colony fragment differed significantly by diet (ANOVA: p < 0.001, F_5,42 _= 13.338). A posthoc (Tukey-HSD) test revealed that worker groups fed diet A (with essential amino acids; AA) raised more pupae than did control groups (diet A mean ± SD: 40 ± 9.02, controls: 29.63 ± 15.17 pupae), albeit not significantly more (Figure 1). With essential AA present in the diet, treatment with the antibiotic Rifampicin to reduce endosymbiont numbers produced no significant effect (diet AR: 24.5 ± 8.8 pupae), just as did omission of essential AA from the diet (diet B: 30.13 ± 14.6 pupae). However, worker groups treated with antibiotics and kept on a diet lacking essential AA raised significantly fewer pupae than did all other groups (diet BR mean ± SD: 4 ± 5.45 pupae) except for workers fed sucrose solution only; the latter produced pupal numbers statistically indistinguishable from those of BR groups (diet S: 8.25 ± 8.66 pupae) (Figure 1).

**Figure 2:**
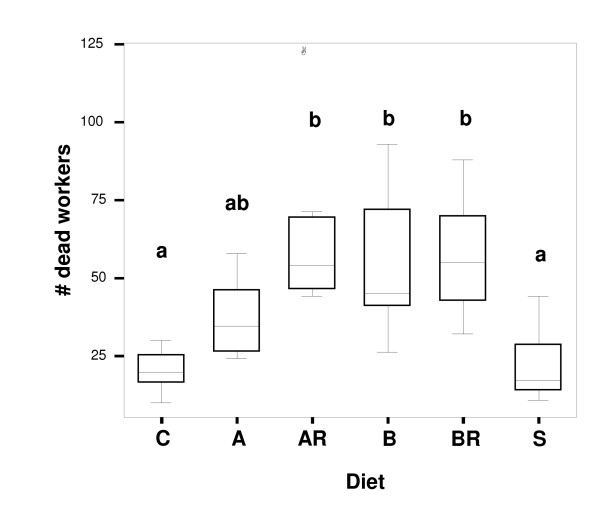
**Comparison of worker mortality across diets**. Numbers of dead workers (of 150 individuals per group initially) after a 12-week experimental period; differences among diet categories were significant (ANOVA: p < 0.001, F_5,42 _= 8.459; see Results). Groups that differed significantly from each other in Tukey HSD posthoc test are marked with different letter above box-plots. Diet A (artificial diet with essential AA) is intermediate between groups and not significantly different from these groups. Diets as in legend of Figure 1. Median is given as line in boxes, outliers are indicated by a circle.

Over the 12-week experimental period, worker mortality differed significantly between feeding groups (ANOVA: p < 0.001, F_5,42 _= 8.459) (Figure 2). Significantly fewer individuals died in control and sugar-only groups than in groups treated with antibiotic (Tukey-HSD test; diet C: 20.38 ± 6.5 dead workers from 150 and diet S: 21.75 ± 11.18, versus diet AR: 63.13 ± 25.51, and diet BR: 57 ± 18.63 dead workers) or those whose diet lacked essential AA (diet B: 54.38 ± 24.67 dead workers). Worker groups fed diet A (with essential AA) showed intermediate levels of mortality (37 ± 18.63 dead workers) (Figure 2). Mortality was not source colony-specific (ANOVA: p = 0.304, F_7,40 _= 1.21) and not significantly correlated with the ability of worker groups to raise brood (Pearson's Product Moment Correlation Coefficient *r *= -0.158, n = 48, p = 0.284). Thus, brood production per worker group depended solely on diet, irrespective of the number of workers dying per group during the experimental period.

**Figure 3:**
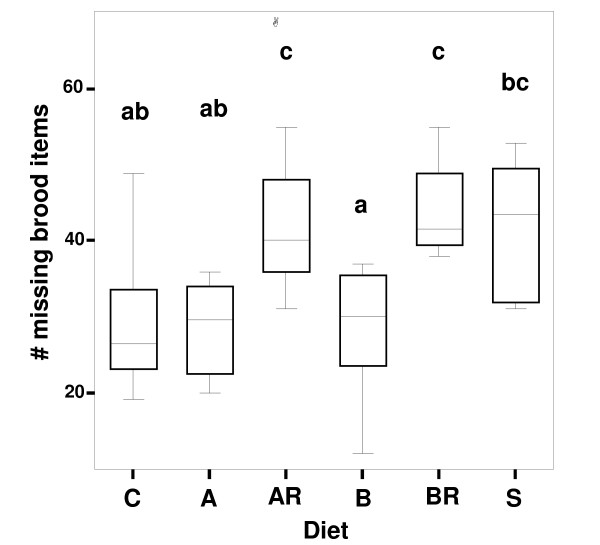
**Comparison across diet categories of numbers of brood items missing**. Number of brood missing from a total of 90 eggs and first instar larvae distributed to each worker group over the 12-week experimental period. Differences across diets were significant (ANOVA: p < 0.001, F_5,42 _= 6.287; see Results). Groups differing significantly from each other in a Tukey HSD posthoc test are marked with different letters above the boxes. Diet categories marked with two letters are intermediate and not significantly different from the respective groups. Diets as in legend of Figure 1. Lines within boxes represent medians, and an outlier is indicated by a circle.

Brood may be eaten by workers either when damaged or when food is too limited [29]. For each worker group, we added the number of pupae produced and removed by us to the number of larvae still present, and subtracted this from 90 to quantify the number of brood items lost during trials. After 12 weeks, groups differed significantly in number of brood items missing from the total of 90 items supplied during the experiment (ANOVA: p < 0.001, F_5,42 _= 6.287). Missing brood were fewest in worker groups fed with diet B (without essential AA), intermediate in groups fed with the control diet, diet A (with essential AA), or sucrose (Tukey-HSD posthoc test), and greatest in groups fed diets AR and BR (artificial diet with and without essential AA and treated with antibiotic) (Figure 3).

**Figure 4:**
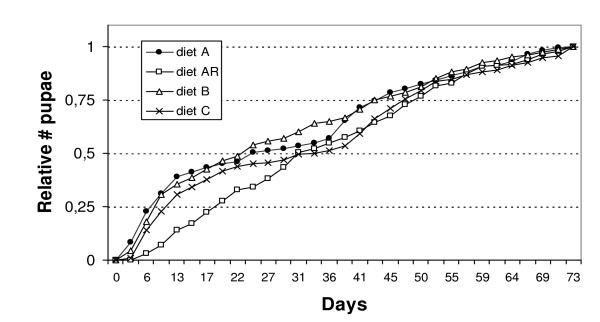
**Brood production across diet categories over time**. Comparison of mean number of pupae produced over time for diets A (artificial diet with essential AA), diet AR (A plus antibiotic), diet B (A minus essential AA) and control (honey water, Bhatkar agar and cockroaches). Numbers of pupae produced are standardized relative values with minimum 0 when no pupae were present in the colony and the maximum number of pupae as 1. A logistic trend analysis revealed that worker groups fed diet AR were significantly slower in the production of pupae compared to groups on the three other diets.

While total production of pupae did not differ significantly between diets A, AR, B, and C, we compared rates of pupal production over time for these diet categories using a logistic trend analysis. Colonies producing fewer than 20 pupae over the experimental period (one control group and one group fed diet AR) were outliers and thus omitted from the analysis. Production rates were standardized by dividing the number of pupae produced for each point of time by the final number of pupae in each group (Figure 4), thus yielding values between 0 (no pupae yet at the beginning of the experiment) and 1 (average maximum number of pupae produced per diet). These standardized production rates were fitted to a logistic function with two parameters (intercept and slope). The 95% confidence intervals were overlapping for all intercepts (fitted to zero), but for the slope, the confidence interval for diet AR did not overlap with those of the remaining groups. The shallower slope for AR (95% confidence limits (10.9; 20.0), versus diet A (3.515; 6.92), diet B (3.778; 7.121), and diet C (4.57; 9.561)) indicates longer development times for groups fed diet AR. Worker groups on diets A and B were able to raise 50% of the pupae within 22 to 27 days, whereas control groups and groups on diet AR need approximately 31 days to raise 50% of the maximum number of pupae (Figure 4). However, in control groups, the first quarter of all pupae produced is reached faster than in groups fed with diet AR. All groups produced brood faster in the first 6 weeks of the experimental period than thereafter.

### Reduction of *Blochmannia *by antibiotic treatment

**Figure 5:**
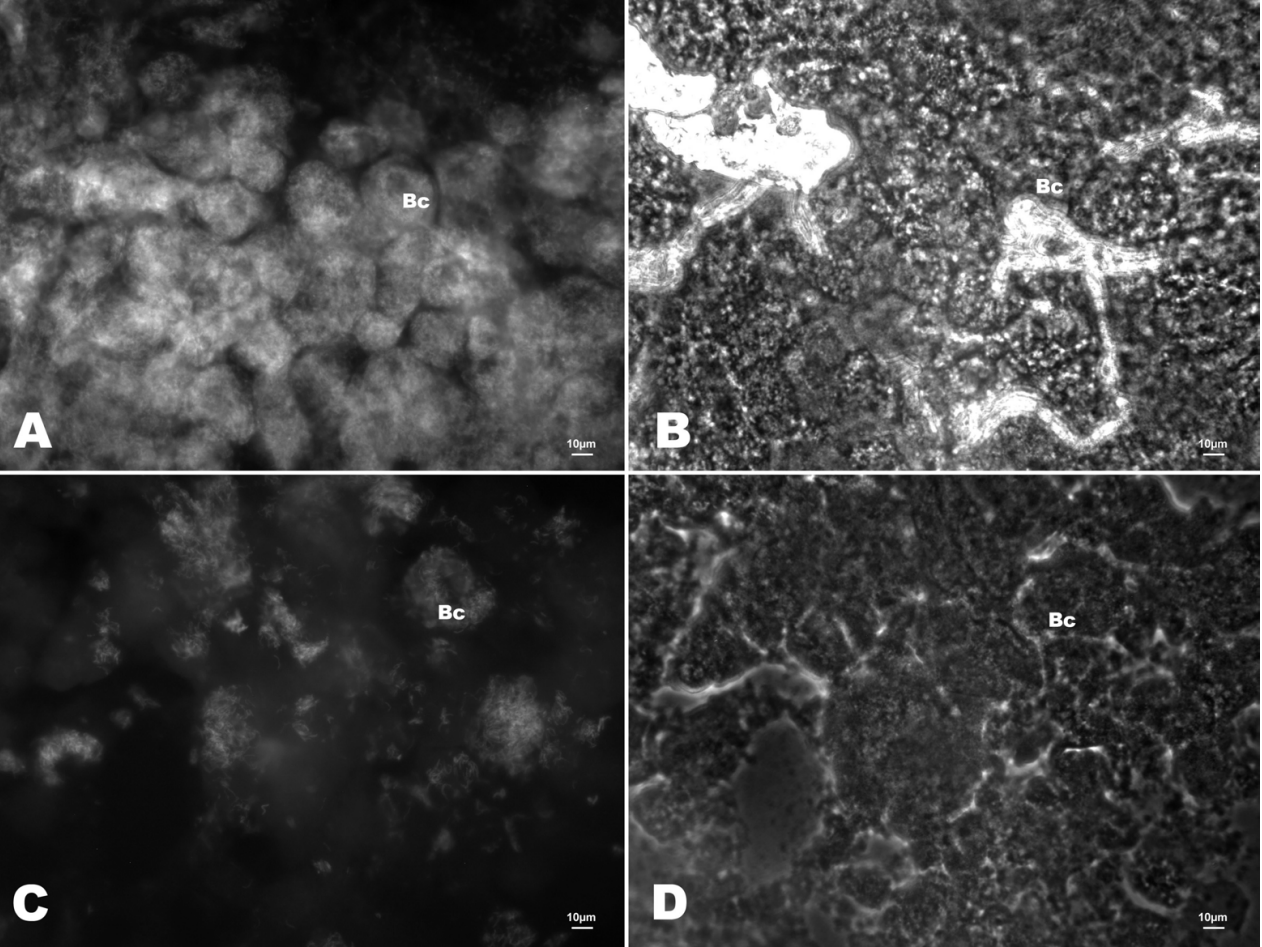
**Efficacy of antibiotic treatment verified by FISH**. *Blochmannia *specific fluorescent in situ hybridisation (FISH) of bacteriocytes (Bc) in the midgut epithelium of: (A) and (B) an untreated worker of *C. floridanus*, and (C) and (D) a worker treated with the antibiotic rifampicin and fed an artificial diet containing all essential amino acids (fluorescence and phase contrast microscopy of the same region, respectively). The midguts of the untreated and the treated worker differ strongly in the number of visible (bacteria containing) bacteriocytes and the number of bacteria per cell. Epithelial gut tissue and other structures visible in phase contrast show no significant autofluorescence. Scale bar 10 μm.

**Figure 6:**
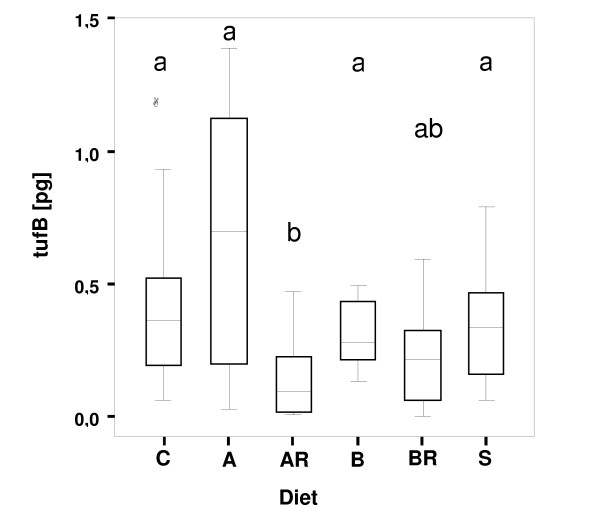
**Efficacy of antibiotic treatment measured by quantitive real-time PCR**. Number of endosymbionts present in the workers after the experimental period measured as pg DNA of the amount of the housekeeping gene *tufB*. Workers differed significantly in their content of *Blochmannia *(ANOVA: Welch- test of square-root transformed data: p < 0.001, F_5,42 _= 5.77). Groups differing significantly from each other in a Tamhane posthoc test are marked with different letters above the boxes. Diet categories marked with two letters are intermediate and not significantly different from the respective groups. Diets as in legend of Figure 1. Lines within boxes represent medians, and an outlier is indicated by a circle.

Reduction of *Blochmannia *numbers in midgut bacteriocytes after treatment with antibiotics was monitored using two different methods: real-time quantitative PCR and Fluorescent *in situ *hybridization (FISH). As qRT-PCR may also detect residual DNA from dead bacteria, FISH was performed to evaluate the amount of living bacteria by targeting the ribosomal RNA. Both techniques revealed significant differences among diets in endosymbiont numbers after the 12-week experimental period (FISH, Figure 5; qRT-PCR: Figure 6). The unspecific eubacterial 16s rDNA probe and the *Blochmannia*-specific probe yielded congruent pictures of the bacteriocytes. Quantitative real-time PCR revealed that workers differed significantly in their content of *Blochmannia *measured as pg of the gene *tufB *encoding an elongation factor (ANOVA: Welch- test of square-root transformed data: p < 0.001, F_5,42 _= 5.77). Comparisons among groups (Tamhane posthoc test) revealed that numbers of endosymbionts were significantly reduced under diet AR (median: 0.094 pg *tufB*/worker) compared to those for all other diets (median: for diet A: 0.695, diet B: 0.280, diet C: 0.366, sucrose: 0.339 pg *tufB*/worker) except BR (median: 0.216 pg *tufB*/worker), the other diet with antibiotics. Numbers of *Blochmannia *in BR feeding groups were lower than those in all groups on diets lacking antibiotics, but significantly so only compared to diet A that had the highest *tufB *content of all groups.

### Feeding experiments with ^15^N-labelled urea

To investigate whether the urease of *Blochmannia *is functional and facilitates N-incorporation into the amino acid pool of the host, we fed ^15^N-urea to the ants. Analysis of ^15^N-enrichment of free amino acids in the hemolymph of workers by gas chromatography-mass spectrometry (GC-MS) revealed significant labelling of several amino acids that could be unequivocally identified (see Methods section for details). Except for the branched-chain, essential amino acids valine, leucine and isoleucine, all amino acids analysed (including the non-essential amino acids alanine, glycine, proline, aspartic acid and glutamic acid as well as the essential amino acids methionine and phenylalanine) showed significantly elevated levels of heavy isotope incorporation; i.e. the naturally occurring level of the heavy isotope of the respective amino acid (M+1) showed no overlap with the mean level and range of standard error in experimental groups (Table 1). Labelling of hemolymph amino acids depends both on the pool size of pre-formed, unlabelled amino acids and turnover as well as the ^15^N-incorporation rate from ^15^N-urea. Therefore, amino acid pools became differentially labelled with ^15^N. Increase in levels of ^15^N-labelled amino acids was stronger for non-essential amino acids (between 4 mol % for glycine to 9 mol % for alanine) than for the essential amino acids methionine (3 mol %) and phenylalanine (3 mol %).

**Table 1:** ^15^N-labelling of free amino acids in the hemolymph of ants fed with [^15^N] urea

	**Calculated natural abundance**	**Urea feeding experiments (control)**		**[^15^N]Urea feeding experiments (labelling)**	
	**M+1 abundance (fraction %)**	**M+1 abundance (fraction %)**	**[^15^N]AA (mol %)**	**M+1 abundance (fraction %)**	**[^15 ^N]AA (mol %)**

**Gly**	7.6	6.8 ± 0.3	-0.8 ± 0.3	11.7 ± 0.8	4.2 ± 0.8
**Ala**	8.5	7.8 ± 0.6	-0.7 ± 0.6	16.7 ± 1.8	8.2 ± 1.9
**Glu**	11.1	10.4 ± 0.3	-0.8 ± 0.3	20.1 ± 2.5	8.9 ± 2.5
**Asp**	10.3	9.6 ± 0.2	-0.7 ± 0.2	16.1 ± 1.7	5.8 ± 1.7
**Met**	10.4	10.8 ± 1.1	0.4 ± 1.1	13.4 ± 0.9	3.0 ± 0.9
**Phe**	12.5	11.8 ± 0.3	-1.7 ± 0.3	16.4 ± 1.2	2.9 ± 1.2

After feeding experiments, free amino acids (AA) from hemolymph of 4–6 ants were derivatized and analyzed by GC-MS. The abundance of the first isotope peak, i.e. the amount of naturally occurring AA containing a heavy isotope (either ^13^C or ^15^N; M + 1) was determined from the mass spectra of amino acids, and additional ^15^N-incorporation (mol %) was calculated. Both non-essential and essential (bold) AA were found to be labelled. Values are obtained from at least three independent experiments and relative amount averaged over samples (mean ± SE).

### Examination of gut microflora of *C. floridanus *by community fingerprinting

Bacterial community fingerprinting by TGGE revealed no gut microbiota other than *Blochmannia *in dissected guts. Additional bands that were detected in whole ant extracts in some instances were only found on thorax controls but never in gut preparations. Cultivation attempts from *C. floridanus *midgut contents yielded a low number (1–20 clones per plate) of *Serratia marcescens *(accession number DQ117670), a known facultative insect pathogen, in two out of three examined ant colonies. FISH analysis of *C. floridanus *midguts with *Blochmannia*-specific and universal 16S rDNA probes was congruent and did not reveal any secondary microbiota.

## Discussion

Our results strongly support the predicted nutritional contribution of *B. floridanus *to its host, in spite of *C. floridanus *being an omnivore. Feeding experiments with chemically defined diets show that *Blochmannia *compensated for the absence of essential amino acids in the food source. Thus, worker groups fed a diet lacking all host-essential amino acids raised just as many pupae as did control groups and groups fed diets containing such amino acids. Significantly reduced numbers of endosymbionts in antibiotic treated hosts had no effect on numbers of pupae produced by worker groups as long as essential amino acids were provided with the diet. In contrast, when endosymbionts were strongly reduced and essential amino acids omitted from the food, worker groups were hardly capable of raising any pupae from the eggs and larvae provided. Reduced success may be due to direct effects on larvae, i.e. antibiotic transmitted by workers to larvae via trophallaxis, and its subsequent effect on endosymbionts within the larvae themselves. As a consequence, larval development may be inhibited by lack of essential nutrients normally provided by *Blochmannia*. Additionally, nutrient content of food regurgitated from the worker crop and transmitted to the larvae may differ between symbiotic and aposymbiotic workers. Zientz and Beyeart et al [19] showed that such indirect, worker-mediated effects play a role in the larval development in *C. floridanus*.

Reduction of *Blochmannia *in workers treated with the bacteriostatic Rifampicin was not complete, as some bacteria were still visible using FISH as well as in qRT-PCR. The latter may partially be explained by the fact that DNA of the endosymbiont may still be detectable by qRT-PCR when bacteria are not alive or active any longer but are still present in the midgut epithelium. Workers examined for their endosymbiont content were quite old (at least 3.5 months) when qRT-PCR was performed after the experimental period and only adult workers with a completely darkened cuticle had been used to build the worker groups. As the symbiosis degrades naturally with age of the workers [27] the differences in number of endosymbionts between antibiotic-treated groups vs groups not treated with antibiotic are expected to be small.

*Blochmannia *content differed strongly among groups not treated with antibiotics after the experimental period (Figure 6). The highest number of endosymbionts was present in workers fed diet A that contains essential AA, whereas workers fed with diet B lacking essential amino acids contained fewer than average *Blochmannia*. Thus, the number of endosymbionts present in the midgut may be controlled by the amount of nutrients present in the food of the ant rather than by nutrient need of the host.

As a side effect of the antibiotic treatment extracellular bacteria in the gut lumen may have been damaged. However, their role in nutritional upgrading in *C. floridanus *should be minor as we rarely detected bacteria in the midgut other than *Blochmannia*, which should have been contamination from the migut epithelium. The failure to detect bacteria other than *Blochmannia *in most specimens examined using either community fingerprints by TGGE, cultivation of bacteria from gut contents or FISH suggests that only few bacteria are regularly residing in the gut of *C. floridanus*. In contrast, we were able to detect regular bacterial residents in the guts of several species of *Tetraponera *(Pseudomyrmicinae) as well as *Dolichoderus *(Dolichoderinae) [13] using the same techniques.

Worker mortality was not directly correlated with numbers of pupae raised from the small larvae and eggs provided. Perhaps as side effects of the antibiotic treatment, mortality was significantly higher in worker groups treated with antibiotics (diets AR and BR), as well as in those fed the artificial diet lacking amino acids (diet B). Such an unbalanced diet has been shown to delay or arrest development in other insect larvae [30] due to stress associated with excretion of excess nutrients that may be detrimental. Surprisingly, mortality was very low in worker groups fed sugar solution only. However, many brood items were missing in these worker groups at the end of the experimental period, and most probably were cannibalized [29,31]. Thus, pupae developing in these groups may have been sustained mainly by nutrients recycled from cannibalized brood, whereas sucrose solution and few other nutrients obtained from missing brood seemed to be sufficient for adult workers to survive.

Feeding experiments with ^15^N-labelled urea also support the hypothesis that *B. floridanus *provides its host with essential amino acids. The ^15^N can be traced to non-essential amino acids in hemolymph and also to L-methionine and L-phenylalanine that are essential to insects [32] but should be a product of bacterial metabolism. The fact that the branched-chain amino acids were not labelled does not necessarily mean that they cannot be produced by *Blochmannia *but may indicate a low biosynthesis rate of these amino acids. Notably, the feeding period with ^15^N-urea was only 8 h and the newly formed ^15^N-labelled amino acids were diluted by the pool of pre-existing unlabelled amino acids. As hemolymph amino acids were found to be labelled up to 9 mol %, it can be concluded that an efficient nitrogen transfer from urea into the hemolymph amino acid pool had occurred.

The symbiont *B. floridanus *possesses a complete urease gene cluster and should thus be able to metabolize urea via hydrolyzation into CO_2 _and ammonium. The latter can be fed into the amino acid metabolism by the activity of glutamine synthetase, which is also encoded in the genome of *B. floridanus *[17]. Shetty [33] has reported that *Camponotus compressus *ingests urine (animal or human) and has shown experimentally that the ants are attracted to aqueous solutions of urea. Bird droppings containing uric acid are also collected by ants of genera *Camponotus *and *Polyrhachis *(Feldhaar, unpublished results). In addition, the endosymbiont may enhance nitrogen-recycling within the host.

## Conclusion

At first glance it may be surprising that the omnivorous *C. floridanus *should harbour an endosymbiont that plays a role in nutritional upgrading, i.e. in enhancing the nutritional value of food resources. However, recent studies have shown that several species of the genus *Camponotus*, as well as of closely related genera *Polyrhachis *and *Echinopla*, can be considered as "secondary herbivores". At least in tropical regions, ants of these genera mainly utilize plant-derived resources, either directly via extrafloral nectaries or indirectly via trophobiosis with sap sucking insects [34,35] whereas only a small amount of nitrogen is obtained via predation or scavenging. Unlike symbiotic relationships between bacterial endosymbionts and insects living on food resources consistently lacking certain nutrients (e.g. essential amino acids for sap-feeding insects), *Camponotus *ants may be able to obtain food that contains all needed nutrients by foraging. However, its endosymbiont *Blochmannia *may open a wider array of ecological niches for the host ants, and may enable them to make use of ecological niches otherwise too limited in certain resources for other ants to inhabit. In addition, *Blochmannia *may confer a significant fitness advantage by enhancing competitive ability with other ant species lacking such an endosymbiont. *Camponotus *is superabundant in the canopy of tropical rainforests, where these ants often feed on N-poor plant exudates either directly or indirectly via trophobiosis [9]. The genera *Camponotus *and *Polyrhachis *are extremely successful in species number and distribution, together comprising ~15 % of all described ant species today. Although *Polyrhachis *is restricted to the Palaeotropics, *Camponotus *can be found in all biogeographic regions of the world [8] and diverse habitat types [10]. Acquisition and domestication of *Blochmannia *in an ancestor of the Camponotini approx. 40 MYA [12,14] was likely a key innovation initiating the diversification and evolutionary success of this important group of ants.

## Methods

### *Camponotus floridanus*: sampling sites and culture

Founding queens of *Camponotus floridanus *were collected (by AE) in Florida in June 2003 and 2004 near Tarpon Springs, Pinellas County (28°8'55''N, 82°45'29''W). Colonies were kept in plastic containers (20 × 20 × 10 cm) with plaster nests in a climate chamber at Wuerzburg University (constant temperature of 25°C, 12 h light per day), and were fed twice a week with cockroaches (*Nauphoeta cinerea*), Bhatkar agar [36] and honey water (1:1) *ad libitum*. Prior to their use in experiments, colonies had reached a size of 2000 to 10000 workers.

### Feeding experiments (antibiotic treatment accompanied by chemically defined diet)

From each of eight large queenright colonies of *C. floridanus*, six groups of 150 randomly picked minor workers were transferred independently into separate artificial nests. Each of the six worker groups was fed three times per week, but on different diets:

• **Control**: Bhatkar agar, cockroaches and honey water *ad libitum*.

• **A**: a cube (0.5 cm sidelength) of chemically defined diet containing all trace metals, vitamins, growth factors and amino acids (AA) essential for the ants (for exact composition and preparation of the diet see [28].

• **AR**: same as A but with 100μl of a solution containing 2% of the antibiotic Rifampicin mixed into the food every other week.

• **B**: chemically defined diet like A except that AA essential for the host were omitted and compensated by higher amounts of non-essential AA, so that the total amount of AA in the diet was not changed.

• **BR**: chemically defined B-diet mixed with 100μl of a 2% Rifampicin-solution every other week.

• **S**: water containing sucrose *ad libitum *(20% w/v) only.

Each worker group was provided with 15 eggs and 15 larvae (1 mm length) at the beginning of the experiment, as well as after 4 and 8 weeks (45 eggs and 45 larvae in total). Over a period of 12 weeks the success of the workers in raising pupae from the early stage brood was monitored. Worker groups were checked three times a week for new pupae as well as for dead workers, and both were removed to prevent supplementation of the worker-diet through cannibalism. As dead workers may already have been disassembled or cannibalized by nestmates, they were counted by the number of head capsules found in the nest. Dead workers were not replaced, and thus the number of workers within each group declined over the experimental period. At the end of the experimental period, all brood members still present in the colony were counted in order to calculate the amount of brood lost during the experimental period. When food is limited or brood items are damaged, brood may be eaten by workers [29,31] and thus the nutritional value of these items would be recycled.

### Real time qPCR

DNA was isolated from whole gasters (including guts and bacteriocytes) of two workers from each group of the six different feeding regimes (see Feeding experiments section). Whole ants were cooled on ice first, and then gasters were cut off with fine scissors. Gasters were homogenized after shock-freezing with liquid nitrogen in an Eppendorf Cap. DNA extraction was performed with a commercial kit (Gentra Systems Puregene^®^, Minneapolis, MN, USA) according to the manufacturer's recommendations. The DNA pellet was resuspended in 50μl double distilled water. For each reaction of real time qRT-PCR, 100 ng total DNA were used as template, and a fragment of approx. 150 bp of tufB (see primer sequence in [19]) was quantified; this exemplary housekeeping gene of *B. floridanus *encodes an elongation factor. C(t) values of the samples were transformed to pg of tufB by using 5, 10, 40 and 100 pg of a purified tufB PCR product obtained with the same primers as a standard template in each run. For each sample, two independent qRT-PCRs were performed using a DNA Engine Opticon System (MJ Research, Waltham, MA, USA) and the ABsolute QPCR SYBR Green Mix (ABgene, Hamburg, Germany). All reactions were performed according to the manufacturers' instructions. The pg *tufB *measured by qRT-PCR were square-root transformed before the statistical analysis.

### Fluorescent *In Situ *hybridisation (FISH)

To determine the success of the antibiotic treatment, bacteriocytes were visualized by FISH with oligonucleotide probes Eub338 [37]), targeting a conserved region of the eubacterial 16S rRNA, and with Bfl172 (5'-CCTATCTGGGTTCATCCAATGGCATAAGGC-3'), targeting a 16S rRNA region specific for *B. floridanus *and the closely related *B. rufipes*. Probes were labelled with the fluorescent dyes Cy3 or FITC at the 5' end by the manufacturer (Metabion, Planegg-Martinsried, Germany). Probe Bfl172 was designed using the software Primrose [38]. Specificity of the probes was checked with RDP II Probe Match [39].

Midguts of *C. floridanus *workers from control groups and groups fed with diet A (containing all essential AA) and treated with antibiotics (2% Rifampicin w/w mixed into the food) were dissected in sterile PBS (pH 7.2). Gut contents were removed prior to fixation on microscopic slides for 2 h in 4% (w/v) paraformaldehyde in PBS. Samples were dehydrated by subsequent 3-min washes in 50%, 70% and 100% ethanol. After air-drying, they were covered with 40μl of hybridisation buffer (35% formamide, 900 mM NaCl, 20 mM Tris/HCl pH 7.5, 0.2% SDS and 2 ng/μl of each fluorescently labelled oligonucleotide probe) and incubated for 2 h at 46°C in a humid chamber in the dark. After hybridisation, the slides were washed for 30 min in 70 mM NaCl, 20 mM Tris/HCl pH 7.5, 5 mM EDTA, 0.01% SDS at 48°C. Samples were rinsed in sterile distilled water, air-dried, mounted in 2 μl VectaShield^® ^(Vector Laboratories Inc., Burlingame, CA, USA) and analyzed with a Leica DMR microscope (Leica Microsystems, Wetzlar, Germany). Photographs were taken with a RT Slider digital camera (Diagnostic Instruments Inc., Sterling Heights, MI, USA) and the Meta Imaging Series 5.0 (Universal Imaging, Downington, PA, USA).

### Feeding experiments with ^15^N-labelled urea

From four different colonies of *C. floridanus*, two groups of 30 workers each (10 minors and 20 majors) were isolated and placed into artificial nests. After a hunger period of 3 days, during which all groups had access to water *ad libitum *but were not fed, control groups were fed honey water (50% w/v) containing 1% urea (Roth), and experimental groups were fed with honey water (50% w/v) with 1% ^15^N-labelled urea (Sigma Aldrich, Taufkirchen, Germany). Workers were anesthetized using CO_2 _8 h after first access to the honey water. Each worker's gaster was removed to prevent contamination of the hemolymph sample with crop contents. The worker's head was then severed from the alitrunk and hemolymph was collected with a 2 μl microcapillary tube from the posterior opening of the head capsule, as well as from the anterior opening of the alitrunk. From each group of workers, approximately 6 μl of hemolymph were collected and frozen (-20°C) immediately after collection.

### Amino acid analysis from ant hemolymph by gas chromatography-mass spectrometry (GC-MS)

Hemolymph (10 μl) was diluted with 50 μl of double distilled water, centrifuged (1000 *g *for 30 sec), and the supernatant was saved. The pellet was extracted twice with 50 μl of double distilled water, and the combined water phases (150 μl) were treated with 30 μl of methanol, 20 μl of pyridine and 30 μl of *N*-propyl chloroformate. After a 5-min incubation time, the mixture was extracted twice with 500 μl of chloroform. The organic phases were taken to dryness under a stream of nitrogen. The residue (*N*-propyloxy-carbonylamide, methyl ester derivatives of amino acids) was reconstituted in hexane for GC-MS analysis. Some of the amino acids could not be efficiently derivatised and therefore escaped detection. Amino acids that could not be measured were all amino acids with additional amide (Asn, Gln), hydroxy (Thr, Ser, Tyr), thiol (Cys) and amine (Arg, Lys) groups in the side chains as well as His and Trp.

GC-MS was performed using a Varian 3400 gas chromatograph interfaced to a Finnigan MAT quadrupole SSQ 700 mass spectrometer. Amino acid derivatives were analyzed on a 25 m, 0.25 μm film thickness Optima-5 column (Macherey and Nagel, Düren, Germany). The injector of the splitless mode was set at 300°C. The GC column temperature was programmed from 80°C to 300°C at 10°C/min. The MS source was set at 150 °C and the electron energy at 70 eV. Compounds were analysed in the positive chemical ionisation mode using isobutane as reactand gas.

### Bacterial community fingerprint of *C. floridanus *gut-microflora

To assess the bacterial gut microbiota in *C. floridanus *the guts of workers from four different colonies were dissected in isolation buffer (35 mM TrisCl, pH 7.6, 25 mM KCl, 250 mM sucrose). Five mid- and hindguts, midgut contents and thoraxes respectively, were pooled for DNA extraction from each colony, followed by universal 16S rDNA PCR and temperature gradient gel electrophoresis (TGGE) as previously described [13].

For analysis of cultivable bacterial species guts from workers of three colonies were homogenized in 50 μl Luria Bertani (LB) medium, plated on LB agar plates and incubated overnight to several days at 30°C and 37°C.

### Statistical analysis

Statistical analyses were performed either with Statistica 7.0 or with SPSS 12.0 (SPSS GmbH). The logistic trend analysis to compare development time between feeding regimes was performed with S-Plus (SAS Institute).

## Authors' contributions

HF and RG planned and coordinated the study and wrote the manuscript. HF fed the labelled urea and collected the hemolymph for GC-MS analysis. MK and MM conducted the GC-MS analysis of hemolymph samples. JS and KB conducted the experiments with the chemically defined diet. KB and SS both conducted the qRT-PCR and SS prepared the FISH slides.
